# The double-edged sword effect of AI technological disruption awareness on design innovation competence: the mediating role of regulatory focus and the moderating role of AI critical thinking

**DOI:** 10.3389/fpsyg.2026.1883577

**Published:** 2026-10-20

**Authors:** Xiang Wang, JianJun Hou, Tingyun Luo, Jia Du, Zilong Yuan

**Affiliations:** 1 School of Art and Design, Nanjing Institute of Technology, Nanjing, China; 2 School of Design, Fujian University of Technology, Fuzhou, China

**Keywords:** AI critical thinking, AI technological disruption awareness, design education, design innovation competence, double-edged sword, regulatory focus theory

## Abstract

**Introduction:**

While the rapid advancement of AI technology is empowering design practice, it is also having a significant impact on the development of design innovation education. In the context of design education, how students' perceived “AI technological disruption awareness (AITDA)” influences their design innovation competence has become a critical issue. Existing research has largely focused on the negative effects of technological disruption, overlooking the complex psychological mechanisms that may be triggered within design learning environments.

**Methods:**

Drawing on regulatory focus theory (RFT), this study examines how students' AI technological disruption awareness (AITDA) is associated with their design innovation competence (DIC) through two parallel motivational pathways, promotion focus (ProF) and prevention focus (PreF), and whether AI critical thinking (AICT) moderates these pathways. A questionnaire survey was administered to 568 design students from Chinese universities, and the moderated dual-path model was tested with confirmatory factor analysis, hierarchical regression, and bootstrap-based mediation and moderated-mediation analyses.

**Results:**

The results show that AITDA was significantly positively associated with DIC, both directly and through the positive pathway of ProF, whereas it was simultaneously significantly negatively associated with DIC through the negative pathway of PreF; the two indirect pathways were comparable in magnitude and opposite in direction, resulting in a non-significant total indirect effect. AICT strengthened the positive AITDA–ProF association and unexpectedly strengthened rather than weakened the AITDA–PreF association; conditional indirect effects and the index of moderated mediation confirmed that the mediating effects of both ProF and PreF varied significantly as a function of AICT.

**Conclusion:**

These findings deepen our understanding of the psychological mechanisms of design innovation under AITDA, highlight the core value of cultivating AICT in design education, and provide a theoretical foundation and practical insights for guiding students toward positive adaptation and innovation amid technological change. However, because the study is based on cross-sectional, self-report data collected at a single point in time, the results should be interpreted as associations rather than causal relationships; longitudinal and experimental designs are needed to establish causality.

## 1 Introduction

With the rapid development of emerging technologies, such as AI, digitalization, and intelligent technologies, these innovations have permeated every industry, placing new demands on the cultivation of innovative talent. In the field of design education, fostering students' innovative capabilities has become crucial for adapting to future societal developments (Hu et al., 2021). Due to its efficiency, precision, and powerful information processing capabilities, AI provides new tools and possibilities for design practice, while simultaneously challenging traditional design processes and the DIC framework (Wang H. Y. et al., 2025; Wang X. et al., 2025).

Research by Xu (2025) indicates that the application of AI technology in image generation, scheme optimization, and user analysis has significantly influenced the content and methods of design work, with certain repetitive and routine design tasks facing the risk of being replaced by automated tools. Against this backdrop, design students, as future design professionals, must not only master new technologies but also address the potential impacts of AI-driven technological change at the cognitive and psychological levels (Yang et al., 2025). This “AITDA,” that is, students' perceptions and concerns regarding AI potentially replacing or reshaping design work, may have complex effects on their innovative motivation, usage behavior, and ultimately, their DIC.

Existing research on the antecedents of individual innovation has largely focused on organizational factors such as leadership styles (Li and Jin, 2025), work environments (Li et al., 2025), and psychological empowerment (Yang et al., 2022). In higher education—and particularly in design education—there is still limited evidence on how psychological perceptions triggered by external technological change relate to the development of students' innovative capabilities. Moreover, previous studies have frequently treated AI technology as a uniformly negative variable, positing that it induces anxiety, self-doubt, and professional-identity crisis and thereby inhibits innovative behavior. For example, Jussupow et al. (2022) found that many medical students and professionals hold negative views of AI because it threatens their professional identity, and Chung et al. (2025) reported that AI awareness is associated with heightened job insecurity and withdrawal-related behavioral outcomes. However, a growing body of recent empirical work paints a more complex picture. Chen et al. (2024) demonstrated that AI anxiety is positively associated with students' motivated learning when accompanied by AI self-efficacy, and Ji et al. (2025) found that human–machine co-creation can simultaneously enhance learning performance and AI awareness. In the design-education domain specifically, Hwang and Wu (2025) showed that generative AI influences design students' creative cognition through a chain mediation of self-efficacy and anxiety; Rong et al. (2025)) systematically documented both empowering and concerning effects of generative-AI art applications in higher education; and Yang et al. (2025) reported a nuanced relationship between AI dependence and college students' innovation capability. These inconsistent findings suggest that the mechanisms linking AI-related perceptions to learning and innovation outcomes are more complex than a simple positive or negative framing, and that the double-edged nature of AI disruption deserves systematic theoretical and empirical treatment in design education.

Therefore, the underlying mechanisms through which AITDA either inhibits or promotes students' DIC remain to be thoroughly elucidated. The RFT (Higgins, 1997) offers a useful perspective for understanding this potential dual effect. This theory posits that individuals exhibit two distinct self-regulatory tendencies when pursuing goals: ProF focuses on growth, achievement, and positive outcomes, often adopting proactive strategies; PreF, on the other hand, emphasizes safety, responsibility, and loss avoidance, typically employing cautious and conservative strategies. In recent years, numerous studies have utilized this theory to explain similar “double-edged sword” effects. For example, Shi et al. (2026) developed a dual-path model based on the RFT to examine how the application of artificial intelligence influences the innovative capabilities of employees in the hospitality industry, exploring the underlying mechanisms and boundary conditions; Zhu et al. (2025), on the other hand, applied this theory to analyze the mediating roles of the Promotion and PreF orientations in the impact of perceptions of technological disruption on the craftsmanship of manufacturing workers.

In the context of this study, when students perceive the impact of AI technology, they may develop different regulatory focuses: some students may view it as an opportunity to enhance their own competitiveness, thereby forming a promotion-oriented tendency; others may focus more on the risks of being replaced or eliminated, and consequently adopt a defensive stance. These two distinct psychological pathways are likely to mediate the differential effects of AITDA on DIC. Furthermore, differences in individual critical thinking abilities (Li et al., 2015; Liu et al., 2023) may moderate the strength of the aforementioned relationship. As a key higher-order thinking and cognitive disposition, AICT is likely to influence students' interpretation of and response to the impact of AI. Therefore, exploring the moderating role of AICT helps reveal the boundary conditions underlying the “AITDA–regulatory focus” relationship.

Based on the above analysis, to clarify the specific impact pathways and boundary conditions of AITDA on students' DIC, this study takes design majors in Chinese universities as the research subjects. Starting from the individual perceptions of design students, it explores the mechanisms through which AITDA influences their DIC. The core research questions addressed in this study include: What kind of impact does AITDA have on students' DIC? Is this impact mediated by ProF or PreF? How does AICT function as a moderator in the “disruption awareness → regulatory focus → DIC” pathway? Through empirical analysis of these questions, this study aims to elucidate the psychological mechanisms underlying the formation of students' DIC in the context of AI technological disruption, thereby providing a theoretical foundation and practical guidance for design education reform and the cultivation of innovative talent.

## 2 Literature review

### 2.1 AI technological disruption awareness

The perception of technological disruption reflects employees' perception of the likelihood that their jobs will be replaced by emerging technologies, including smart technologies, artificial intelligence, robotics, and algorithms (Brougham and Haar, 2018). As AI technology becomes deeply integrated into design practice, its impact has expanded from the tool level to the cognitive and value levels, creating a structural impact that profoundly deconstructs and reconstructs design students' professional perceptions, skill positioning, and creative self-identity. Therefore, in the context of this study, “AITDA” specifically refers to the psychological state formed when design students realize that AI technology may replace certain design capabilities and reshape industry rules and evaluation standards. This state encompasses threat perception, adaptation anxiety, and cognitive adjustment, reflecting their complex psychological responses to technological change.

Current literature primarily explores the impact of AITDA on employee psychology and behavior from the perspectives of organizational behavior and psychology, and generally identifies a dual-path mechanism: on the one hand, AITDA may trigger negative outcomes such as job burnout (Kong et al., 2021), job insecurity (Chung et al., 2025), and turnover intentions (Li et al., 2019); on the other hand, it can also stimulate adaptive coping behaviors in individuals, such as active learning (Zhu and Wu, 2026), skill reshaping, and role reconfiguration (Ji et al., 2025), exhibiting a dialectical characteristic of “coexisting threats and challenges.”

However, within the specific context of design education, discussions regarding how AITDA influences students' AI usage patterns and the development of their innovative capabilities remain limited, particularly lacking in-depth analysis of its underlying psychological mechanisms and boundary conditions. In light of this, this study situates AITDA within the technosocial context of design education and attempts to explore, from a dialectical and process-oriented perspective, the potential “double-edged sword” effect it may exhibit in students' AI usage behaviors and design innovation processes.

### 2.2 Focus regulation theory

RFT, proposed by Higgins (1997), provides an important theoretical framework for understanding differences in self-regulation among individuals when faced with goals and external stimuli. The theory posits that regulatory focus is the way in which individuals regulate pleasure and pain, exerting a systematic influence on their cognition, emotions, and behavior. Regulatory focus can be divided into two basic types: Promotion-Focus and Prevention-Focus. The promotion-oriented focus uses the “ideal self” as a reference point, focusing on growth, gains, and achievements. Its behavioral strategies tend toward proactivity and exploration, it is sensitive to opportunities, and its motivation stems from the pursuit of positive outcomes. The prevention-oriented focus, on the other hand, is guided by the “ought self”, emphasizing safety, responsibility, and obligations. Its behavioral strategies lean toward caution and avoidance, it is more sensitive to threats and losses, and its motivation lies in avoiding negative outcomes. The two types exhibit significant differences in cognitive preferences, emotional responses, and behavioral mobilization, collectively shaping how individuals interpret and respond to changes in the external environment (Tumasjan and Braun, 2012). Scholars have applied RFT to examine work performance (Zhang et al., 2026), employee innovative behavior (Dong et al., 2025), work-family conflict (Ling et al., 2026), and corporate green innovation preferences (Li et al., 2026).

In the context of this study, introducing RFT into the exploration of the relationship between “AITDA” and “DIC” helps reveal the underlying psychological mechanisms of both. This paper argues that when design students face AI technological disruption, their type of regulatory focus (promoting/preventing) systematically moderates the intensity and direction of the effects of “AITDA” on their AI usage behavior and innovation capabilities, thereby constituting a moderated mediation path. This perspective not only helps clarify the boundary conditions of the AITDA effect but also provides a valuable theoretical basis for design education on how to guide students' motivational orientations to encourage them to respond more proactively to technological change.

### 2.3 Design innovation competence

DIC has long been regarded as a core objective and key developmental focus in design education (Brown and Kuratko, 2015). Against the backdrop of rapid changes in today's social and technological environment, this competency is not only relevant to the professional competence of individual designers but also directly impacts the effectiveness of the design discipline in addressing complex real-world challenges (Dixon and Murphy, 2016). Generally speaking, DIC can be defined as the holistic ability of designers to systematically integrate multidisciplinary knowledge, practical skills, creative thinking, and humanistic values when facing open-ended, complex, and ill-defined problem contexts. Through continuous exploration and comprehensive evaluation, designers ultimately propose solutions that embody novelty, feasibility, and sociocultural value (Hu et al., 2021).

In relevant academic discussions, DIC is often deconstructed into several interrelated and mutually reinforcing dimensions, which together form a complete competency chain spanning from problem perception to solution implementation and on to reflection and iteration (Lages et al., 2020). These primarily include: creative problem-solving (Hilliges et al., 2007); concept generation and visualization (Sonalkar et al., 2013); technology integration and implementation (Iansiti and West, 1999); user empathy and insight (Li et al., 2024); and critical reflection and iterative optimization (Chen et al., 2026). These dimensions are not arranged linearly but rather intertwine and synergize in design practice, collectively propelling the design process from its ambiguous early stages toward concrete outcomes. By systematically cultivating these multidimensional competencies, design education aims to equip students not only with the skills required to complete design tasks but also with the comprehensive literacy to continuously innovate in complex environments and responsibly address future challenges. As AI technology is gradually integrated into the design process, the innovative capabilities of design students are undergoing profound changes. The extent to which AI technology promotes or inhibits the innovative capabilities of design students has become a research topic worthy of in-depth exploration.

### 2.4 AI critical thinking

Critical thinking is rooted in a deep tradition of philosophy and pedagogy, and its essence lies in the careful examination and rigorous evaluation of various claims, evidence, and the reasoning processes behind them (Bailin and Siegel, 2003). With the advancement of digital technology, its scope has expanded to encompass “digital literacy” (Tinmaz et al., 2022) and “information literacy” (Al-Azri et al., 2025), emphasizing an individual's ability to identify, understand, evaluate, and create information in digital environments.

However, as AI, particularly generative AI, becomes deeply integrated into the fields of creativity and design, traditional frameworks of digital critical thinking struggle to adequately address the complex cognitive, ethical, and value-based challenges this integration raises (Al-kfairy et al., 2024). AI exists not merely as a technological tool but as an “agent” capable of reshaping creative logic, evaluation criteria, and even social contexts. A series of issues, including the system's inherent “black box” nature, potential algorithmic biases, intellectual property disputes, and the resulting social displacement effects (Wamba et al., 2025), require a more systematic, in-depth, and reflective set of competencies. Against this backdrop, AICT has emerged as an integrative theoretical construct. AICT transcends mere technological use or evaluation of outcomes; it is a multidimensional competency framework that integrates technological critique, ethical assessment, value deliberation, and social contextual awareness.

### 2.5 The double-edged sword effect: definition and evaluation criteria

A central premise of this study is that AITDA may exert a double-edged effect on DIC. Following recent work on paradoxical effects of AI (Dong et al., 2025; Zheng et al., 2025), we define a double-edged sword pattern as one in which (1) the two specific indirect effects are both statistically significant but opposite in sign, and (2) their absolute magnitudes are comparable, such that the total indirect effect is small or non-significant while the direct effect remains significant. Under this definition, the effect is evaluated by comparing the magnitude and proportion of each specific indirect effect relative to the total effect, rather than by the sign of any single coefficient. This operationalization allows us to determine empirically whether the positive and negative pathways form a balanced, offsetting pattern, that is, a genuinely “symmetrical” double-edged sword, or whether one pathway dominates.

## 3 Model development and assumptions

### 3.1 Mechanisms of action of AITDA, ProF, and DIC

ProF individuals are driven by growth, ambition, and the realization of their ideal selves; their cognitive and behavioral patterns are highly sensitive to positive outcomes and potential gains (Scholer et al., 2019). Multiple studies indicate that awareness of technological shocks has a positive impact on ProF (Ma and Zhang, 2025; Wu and Zhang, 2024) .

Within this framework, a high level of AITDA, that is, a profound awareness of the rapid evolution of AI technology and its potential to reshape industry norms and render traditional skills obsolete, does not necessarily trigger anxiety or avoidance. Instead, such individuals are more likely to interpret it as an “opportunity-oriented framework.” Design students tend to view technological disruption as a positive signal of industry upgrading and personal capability advancement, thereby stimulating their intrinsic motivation to actively learn and integrate emerging AI design tools and driving them to explore cutting-edge design models of human-machine collaboration. From the perspective of RFT, this cognitive evaluation process, which reframes external threats as internal motivation, is the key psychological mechanism through which awareness of disruption is transformed into action. Therefore, this study proposes the following hypothesis: H1: AITDA has a significant positive effect on students' ProF.

Furthermore, the inclination toward excellence and focus on growth potential inherent in ProF directly shapes students' goal-setting and strategy selection in design practice, specifically manifesting as a greater tendency to proactively transform the potential of AI technology into novel and effective design solutions (Dai et al., 2026). This achievement-oriented self-regulation model provides sustained motivation and cognitive resources for the flexibility and originality required in design innovation (Wang et al., 2020). Therefore, this study proposes the following hypothesis: H2: ProF has a significant positive effect on students' DIC.

Because ProF channels the motivation generated by disruption awareness into the concrete design activities that produce novel solutions, it functions as the psychological conduit through which AITDA is translated into DIC: disruption awareness first shifts students toward a growth-oriented focus, and that focus subsequently supplies the motivation and cognitive resources for innovation. Accordingly, this study hypothesizes: H3: ProF mediates the positive relationship between AITDA and DIC.

### 3.2 The constraint pathways of AITDA, PreF, and DIC

In contrast to ProF individuals, PreF individuals prioritize fulfilling responsibilities, ensuring safety, and avoiding risks, and are particularly sensitive to potential negative outcomes and threat signals in their environment (Scholer et al., 2019). Research indicates that awareness of technological disruption also has a positive impact on PreF (Shi et al., 2026; Zhu et al., 2025).

In the context of this study, for design students, AITDA of equal intensity may also be activated and interpreted as a “threatening frame”, meaning they are more likely to view AI technological disruption as a potential threat to the value of their existing skills, academic stability, and even future career security. This perception of threat easily induces higher state anxiety and avoidance of uncertainty, thereby reinforcing the individual's risk-averse tendencies. Such psychological and behavioral patterns may not only inhibit adaptive learning triggered by awareness of disruption but also solidify a defensive inertia toward innovation, thereby mechanically blocking the pathway from awareness of disruption to exploratory behavior and exerting direct or indirect negative impacts on innovation performance. In light of this, this study proposes the following hypothesis: H4: AITDA has a significant positive effect on students' PreF.

Furthermore, the “avoidance of error” ogic emphasized by PreF and the heightened vigilance toward negative outcomes will systematically constrain the design innovation process. This tendency reduces tolerance for ambiguity, restricts the breadth and depth of conceptual divergence, and leads to an overreliance on safe options during solution selection, thereby imposing structural constraints on DIC, particularly its breakthrough and originality dimensions. Therefore, this study proposes the following hypothesis: H5: PreF has a significant negative impact on students' DIC.

By the same token, PreF is the conduit for a negative indirect association: disruption awareness activates vigilance and loss avoidance, which in turn narrows conceptual exploration and is associated with lower DIC. The threat appraisal initiated by AITDA is thus carried forward—through the heightened prevention focus—to constrain innovation outcomes. Accordingly, this study hypothesizes: H6: PreF mediates the relationship between AITDA and DIC.

### 3.3 The regulatory role of AICT

In numerous studies, critical thinking is often regarded as a key variable, with its role examined across various contexts. For example, the study by Zhang and Zong (2019) explicitly treated critical thinking as a moderating variable to test its moderating effect on the relationship between “psychological capital” and “failure learning.” Wang and Zhang (2026) examined the impact of four higher-order cognitive processes, including AI-supported critical thinking and metacognition, on writing quality. Zhu and Yang (2026) investigated the effects of AIGC use on graduate students' research capabilities and found that critical thinking plays a positive mediating role in this relationship.

Given the existing literature, this study posits that AICT plays a crucial regulatory role between AITDA and individual regulatory focus. High levels of AICT help students move beyond simplistic, emotional “opportunity/threat” dichotomies in response to technological disruption, fostering a more complex and dialectical perspective. Specifically, for students initially inclined toward ProF, higher AICT enables them to more rationally identify potential risks embedded within opportunities, thereby formulating more strategic learning and innovation plans. This optimizes their proactive behavior rather than merely amplifying it, making ProF more resilient and effective.

Therefore, we propose: H7a: AICT plays a positive moderating role in the relationship between AITDA and ProF; that is, the higher the level of AICT, the stronger the positive influence of AITDA on ProF.

For defensively focused students who are susceptible to threat perception, AICT acts as a “cognitive buffer” (Suryanto et al., 2025). It helps students deconstruct the absoluteness of perceived threats, analyze the specific components, temporal span, and personal controllability of technological shocks, and distinguish between real and imagined threats, thereby weakening the extent to which shock awareness directly and automatically triggers intense defensive reactions (Jia and Tu, 2024; Melisa et al., 2025). This implies that high AICT may partially “neutralize” the threatening interpretation of the sense of impact, mitigating the intensity of its transformation into a PreF.

Therefore, this study hypothesizes: H7b: AICT plays a positive moderating role in the relationship between AITDA and PreF; that is, the higher the level of AICT, the weaker the positive effect of AITDA on PreF.

### 3.4 Research model

This study aims to integrate AITDA, RFT, and AICT to reveal the mechanisms through which these factors influence students' DIC via a moderated double-mediation model, and to further explore their distinct influence pathways. Based on this, the study constructs a research framework encompassing AITDA, ProF, PreF, AICT, and DIC, and proposes the hypothetical model shown in Figure 1.

**Figure 1:**
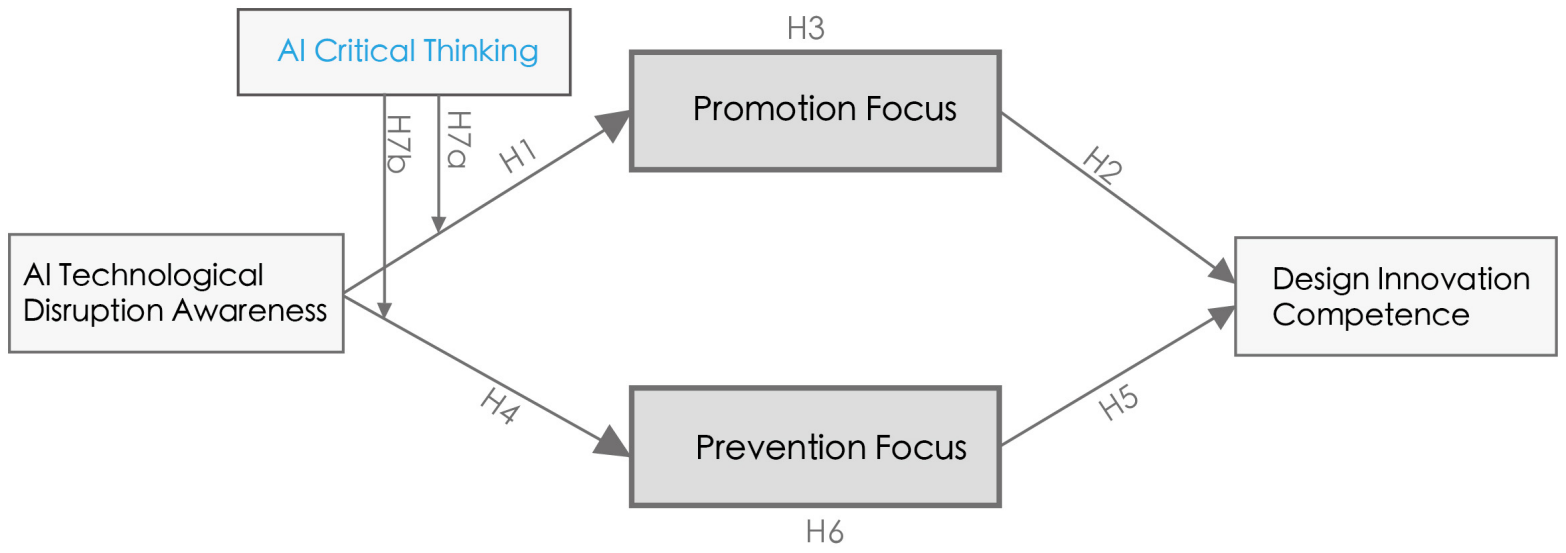
Hypothetical model.

### 3.5 Definition and measurement of variables

The main variables in this research model include AITDA, ProF, PreF, AICT, and DIC. The measurement indicators for these five latent variables are shown in Table 1.

**Table 1:** Variable definitions and measurements.

Variables	Operational definition	Question item	References
AITDA	In this study, this refers to the extent to which individual students perceive the development and application of AI technologies (such as generative AI and intelligent design tools) as posing a potential threat of replacement and a source of uncertainty when completing design innovation tasks.	AITDA1: I'm worried that AI will eventually replace many of the design skills I'm learning now.	Zhu et al., 2025; Brougham and Haar, 2018; Fu and Hou, 2025
		AITDA2: I believe that a significant portion of the design work I'm currently studying and doing will likely be handled by AI in the future.	
		AITDA3: With the rapid advancement of AI, I feel that the value of investing a great deal of effort into learning certain traditional design skills is diminishing.	
		AITDA4: I feel that in the future, the pressure to collaborate or compete with AI in the field of design will only grow.	
ProF	In this study, this refers to students' tendency toward self-regulation when engaging in design innovation activities using or interacting with AI technology, guided by the pursuit of growth enabled by technology, the realization of higher creative ideals, and the attainment of positive innovation outcomes.	ProF1: When using AI for design, I'm always thinking about how to leverage it to bring my creative vision to life.	Higgins, 1997; Maduku, 2024; Crowe and Higgins, 1997
		ProF2: I'm eager to try out all kinds of new AI design tools and methods, and I'm not afraid of the challenges that come with learning.	
		ProF3: I find it incredibly inspiring when designs created with AI receive recognition.	
		ProF4: I often keep track of the progress I've made in using AI for design.	
		ProF5: I always actively seek out opportunities to improve my AI design skills.	
		ProF6: I believe that mastering AI technology will help me achieve greater success in the field of design in the future.	
PreF	In this study, this refers to students' tendency toward self-regulation, guided by the need to avoid errors, mitigate risks, fulfill responsibilities, and meet basic requirements, when engaging in design innovation activities using or interacting with AI technology.	PreF1: When using AI for design, my first priority is to avoid mistakes or undesirable outcomes.	Higgins, 1997; Zhang et al., 2026; Crowe and Higgins, 1997; Xu and Zhang, 2022
		PreF2: I believe it is my responsibility to strictly adhere to relevant ethical and operational guidelines when using AI to complete design tasks.	
		PreF3: I'm worried that if AI is used improperly, it could lead to my designs receiving negative feedback.	
		PreF4: Rather than experimenting with the latest AI technologies, I prefer to use tools that have already been proven to be safe and reliable.	
		PreF5: I'm learning to use AI mainly to meet the basic requirements of my courses or projects, rather than out of personal interest.	
		PreF6: When using AI tools, I double-check my work to ensure there are no omissions or errors.	
AICT	In this study, this refers to the higher-order cognitive abilities that students possess to monitor, evaluate, and adjust their own design thinking processes when facing AI-driven technological disruption and engaging in design innovation activities.	AICT1: When presented with design proposals generated by AI tools, I will first verify their soundness and feasibility rather than accepting them wholesale.	Larson et al., 2024; Darwin et al., 2024; Helal et al., 2025; Gerlich, 2025
		AICT2: I am willing to re-evaluate and adjust my design approach based on new information (including AI suggestions).	
		AICT3: Throughout the design process, I will continually evaluate whether the current approach (including whether and how AI tools are being used) is effective, and make adjustments as needed.	
		AICT4: To create better designs, I will actively seek out and evaluate a wide range of information and perspectives related to AI technology.	
		AICT5:After completing a design phase, I reflect on the entire thought process to identify what went well and what could be improved.	
		AICT6: I often take the initiative to explore the causes and logic behind popular design trends or AI-generated styles.	
		AICT7: When evaluating designs, I am aware of the potential for judgment bias stemming from personal preferences and emotions, and I strive to overcome it.	
DIC	In this study, this refers to students' ability to generate novel, unique, and practical solutions or works during design activities.	DIC1: I can propose novel and unique design concepts or solutions that differ from conventional approaches.	Faridizad et al., 2024; Magistretti et al., 2021; Mendoza-Silva, 2021
		DIC2: My design effectively addresses the target problem and truly meets the core needs of the users, context, or project.	
		DIC3: During the conceptual phase, I can approach the problem from multiple angles to generate a variety of design possibilities or alternatives.	
		DIC4: I can translate creative concepts into concrete, detailed, and actionable design solutions or prototypes.	
		DIC5: My design work is not only aesthetically pleasing in form, but also has a positive impact in terms of functionality, user experience, or social value.	

## 4 Materials and methods

### 4.1 Design and procedure

This study employed a questionnaire survey method to collect data. Prior to the formal survey, a pilot study was conducted among design students in the Nanjing area from November to December 2025 to test the questionnaire's reliability and validity, resulting in a total of 102 completed questionnaires. In the pilot stage, reliability analysis (Cronbach's α and corrected item-total correlations, CITC) and exploratory factor analysis (EFA) were conducted; items with unsatisfactory psychometric properties (e.g., low factor loadings, substantial cross-loadings, or low CITC values) were eliminated. Based on feedback from users in the field of AI design, the wording of the retained items was optimized to minimize complex and ambiguous phrasing, thereby enhancing the readability and consistency of item interpretation. The final scale comprises 28 Likert-type items measuring the five constructs (AITDA, ProF, PreF, AICT, and DIC), each rated on a 7-point scale (1 = strongly disagree, 7 = strongly agree).

The final questionnaire consists of three sections. Section 1 introduces the research context and defines the key variables; Section 2 collects participants' basic information (age, gender, education level, grade, Experience with AI, weekly creation time, and formal AI training, etc.); and Section 3 measures the five constructs with the 28 items described in Table 1.

The formal survey employed a combination of convenience sampling and snowball sampling. Electronic questionnaires were distributed between January and February 2026 to undergraduate and graduate students majoring in design at comprehensive universities and specialized art and design institutions in multiple regions of China. This study was approved by the Research Ethics Committee of the School of Art and Design, Nanjing Institute of Technology (approval number: [NJIT-IRB-20251012-01]). Before participation, all respondents were informed of the research purpose, the voluntary nature of participation, their right to withdraw at any time without consequence, and the way their data would be used; completion and submission of the questionnaire constituted informed consent. The questionnaire did not collect names, student numbers, or other direct identifiers, and all responses were stored anonymously in encrypted files accessible only to the research team. The de-identified data will be retained for a limited period and then destroyed, and no personal information that could identify individual respondents is reported in this manuscript. A total of 632 questionnaires were collected. After excluding invalid responses, 568 valid questionnaires were obtained, resulting in a valid response rate of 89.87%.

In structural equation modeling, the recommended ideal ratio of parameter estimates to sample size is 1:10 (Jackson, 2003). This study involved 28 measured variables and a valid sample size of 568, resulting in a variable-to-sample ratio of 1:20.29, which meets and exceeds the methodological recommendation.

### 4.2 Statistical tools and modeling details

To test the research hypotheses, this study utilized SPSS 24.0 and AMOS 23.0. In AMOS, the measurement model was specified with each of the 28 items loading on its theoretically assigned factor only, with no cross-loadings and no correlated error terms across items of different constructs; the model was estimated with maximum likelihood and compared against theoretically plausible alternative models (four-, three-, two-, and single-factor solutions). For the mediation and moderated-mediation analyses, bootstrap resampling with 5,000 resamples and bias-corrected 95% confidence intervals was used, following the conditional-process framework (Edwards and Lambert, 2007; Hayes, 2018; Preacher and Hayes, 2008; Preacher et al., 2007). Multicollinearity was assessed with the variance inflation factor (VIF); all VIFs were below 3 (Table 2), well below the conventional threshold of 10, indicating no serious multicollinearity.

**Table 2:** Unstandardized regression coefficients, standard errors, and variance inflation factors.

Predictor	ProF model b (SE)	PreF model b (SE)	DIC model b (SE)	DIC model with mediators b (SE)	VIF
Constant	2.6810 (0.3806)	3.5545 (0.4034)	3.0190 (0.3914)	3.8582 (0.3877)	—
Gender	−0.1798 (0.0973)	0.0853 (0.1031)	−0.0944 (0.1000)	−0.0233 (0.0904)	1.02
Age	0.0810 (0.0444)	0.0918 (0.0471)	0.0183 (0.0457)	0.0375 (0.0413)	1.04
Education	0.0272 (0.0915)	0.0082 (0.0970)	0.0502 (0.0941)	0.0477 (0.0847)	2.63
Grade	0.0317 (0.0370)	0.0023 (0.0392)	0.0175 (0.0380)	0.0118 (0.0343)	2.64
Experience with AI	0.0319 (0.0442)	0.0305 (0.0469)	−0.0450 (0.0455)	−0.0397 (0.0410)	1.02
Weekly creation time	−0.0321 (0.0342)	−0.0690 (0.0362)	0.0325 (0.0351)	0.0121 (0.0318)	1.04
Formal AI training	0.0942 (0.0974)	0.0633 (0.1032)	0.0253 (0.1002)	0.0306 (0.0903)	1.02
AITDA	0.4297^***^ (0.0365)	0.2143^***^ (0.0387)	0.3469^***^ (0.0375)	0.3424^***^ (0.0383)	1.32
ProF				0.2086^***^ (0.0393)	1.29
PreF				−0.3934^***^ (0.0371)	1.09
*R* ^2^	0.220	0.073	0.142	0.307	

^*^p < 0.05, ^**^p < 0.01, ^***^p < 0.001.

### 4.3 Common method bias

Because the data were collected with self-report questionnaires, common method bias (CMB) may be a concern. Several procedural remedies were applied before data collection: the survey was administered anonymously, participants were informed that there were no right or wrong answers, completion time was recorded and controlled, some items were reverse-worded, and the questionnaire was administered in separate modules (Zhou and Long, 2004). Statistically, Harman's single-factor test was first conducted: under unrotated principal component analysis, five factors with eigenvalues > 1 emerged, and the first factor explained 31.54% of the total variance, below the 40% heuristic threshold. Second, a latent method-factor test was conducted in AMOS by adding a common method factor to the measurement model (Table 3); the changes in fit indices were negligible (|ΔRMSEA| = 0.000, |ΔSRMR| = 0.0051, |ΔTLI| = 0.001, |ΔCFI| = 0.001; all below 0.01), indicating that the common method factor did not significantly improve model fit. Although both tests suggest that CMB is unlikely to seriously distort the results, the first factor's variance share is not negligible, and the residual risk of CMB is revisited in the Limitations.

**Table 3:** Results of the common method bias test.

Model	χ^2^	df	χ^2^/df	RMSEA	SRMR	TLI	CFI	|Δ RMSEA|	|Δ SRMR|	|Δ TLI|	|Δ CFI|
Measurement model	496.417	340	1.46	0.028	0.0414	0.984	0.986	—	—	—	—
Method-factor model	489.047	313	1.451	0.028	0.0363	0.985	0.987	0.000	0.0051	0.001	0.001

## 5 Results

### 5.1 Descriptive statistics

The demographic characteristics of the 568 valid respondents are summarized in Table 4. The sample exhibited a balanced gender composition, with males accounting for 50.35% and females 49.65%. The majority of participants were young adults aged 18–26 years (78.86%), among which the 18–20 and 21–23 age brackets represented the two largest subgroups. In terms of educational attainment, bachelor's degree holders formed the largest proportion (54.58%), followed by associate-degree (20.42%) and master's degree students (18.31%), while doctoral respondents made up 6.69%. All participants were design majors, covering a full spectrum of undergraduate and postgraduate cohorts.

**Table 4:** Demographic characteristics of the sample.

Characteristic	Category	*n*	%
Gender	Male	286	50.35
	Female	282	49.65
Age	18–20 years old	213	37.5
	21–23 years old	178	31.34
	24–26 years old	141	24.82
	27 years old and above	36	6.34
Education	Associate degree	116	20.42
	Bachelor's degree	310	54.58
	Master's degree	104	18.31
	Doctoral degree	38	6.69
Major Grade	Design	568	100
	Freshman	95	16.73
	Sophomore	107	18.84
	Junior	108	19.01
	Senior	96	16.90
	First-year graduate	38	6.69
	Second-year graduate	36	6.34
	Third-year graduate	50	8.80
	Doctoral and above	38	6.69
	Design-related majors	568	100.00
Experience with AI	Less than 6 months	60	10.56
	6 months−1 year	183	32.22
	1–2 years	241	42.43
	More than 2 years	84	14.79
Weekly creation time	Almost none	132	23.24
	1–5 h	109	19.19
	6–10 h	111	19.54
	11–15 h	107	18.84
	More than 16 h	109	19.19
Formal AI training	Yes	293	51.58
	No	275	48.42
Total	568	100	

With respect to AI experience, respondents were distributed across four duration groups: < 6 months, 6 months−1 year, 1–2 years, and more than 2 years. Weekly creative working time was relatively evenly spread across the sample. A slight majority of respondents (51.58%) had received formal AI training. Overall, this sample encompassed design students with varied educational backgrounds, grade levels, and prior AI exposure.

### 5.2 Reliability analysis

The reliability of the scale was assessed using Cronbach's α and the corrected item-total correlation (CITC). As shown in Table 5, all CITC values exceeded 0.70, and the Cronbach's α of each construct exceeded 0.89; deleting any item did not increase the construct's α. The scale therefore showed good internal consistency.

**Table 5:** Reliability analysis results.

Variables	Name	CITC	The deleted α coefficient	Cronbach's α coefficient
AITDA	AITDA1	0.745	0.877	0.897
	AITDA2	0.782	0.864	
	AITDA3	0.787	0.862	
	AITDA4	0.772	0.868	
ProF	ProF1	0.793	0.908	0.924
	ProF2	0.765	0.912	
	ProF3	0.795	0.908	
	ProF4	0.748	0.915	
	ProF5	0.788	0.909	
	ProF6	0.796	0.908	
PreF	PreF1	0.794	0.898	0.917
	PreF2	0.761	0.903	
	PreF3	0.778	0.9	
	PreF4	0.757	0.903	
	PreF5	0.75	0.904	
	PreF6	0.75	0.904	
AICT	AICT1	0.78	0.922	0.932
	AICT2	0.783	0.922	
	AICT3	0.819	0.918	
	AICT4	0.775	0.923	
	AICT5	0.765	0.923	
	AICT6	0.802	0.92	
	AICT7	0.75	0.925	
DIC	DIC1	0.779	0.866	0.897
	DIC2	0.71	0.882	
	DIC3	0.757	0.871	
	DIC4	0.756	0.872	
	DIC5	0.723	0.879	

### 5.3 Validity

#### 5.3.1 KMO and Bartlett's test

The Kaiser–Meyer–Olkin (KMO) test and Bartlett's test of sphericity were conducted to assess whether the data were suitable for factor analysis (Table 6). The KMO value was 0.934, and Bartlett's test was significant (χ^2^ = 11,096.325, df = 378, *p* < 0.001), indicating that the data were appropriate for factor analysis.

**Table 6:** KMO and Bartlett's test.

KMO value		0.934
Bartlett's test of sphericity	Approximate chi-square (χ^2^)	11096.325
	df	378
	*p*-value	0.000

#### 5.3.2 Variance explained

In this study, principal component analysis was employed to extract factors from all 28 items. Based on the criterion of eigenvalues > 1, a total of 5 common factors were extracted, accounting for 72.401% of the cumulative variance. This indicates that the factor structure is well-represented, effectively explains most of the variance, and yields an overall satisfactory extraction result (as shown in Table 7).

**Table 7:** Explanation of total variance.

Term	Total	Initial eigenvalue			Extraction sums of squared loadings			Sum of squared rotational loadings		
		Variance percentage	Accumula -ted %	Total	Variance percentage	Accumulated %	Total	Variance percentage	Accumulated %
1	8.831	31.541	31.541	8.831	31.541	31.541	5.068	18.101	18.101
2	5.389	19.246	50.787	5.389	19.246	50.787	4.449	15.889	33.991
3	3.011	10.755	61.542	3.011	10.755	61.542	4.259	15.211	49.202
4	1.623	5.797	67.338	1.623	5.797	67.338	3.589	12.816	62.018
5	1.417	5.062	72.401	1.417	5.062	72.401	2.907	10.382	72.401

#### 5.3.3 Rotated factor loadings matrix

To enhance the interpretability of the factors, this study employed the varimax rotation method to rotate the factors, thereby concentrating the variables on a smaller number of factors with higher loadings and more clearly revealing the correspondence between the factors and the study items. The rotated factor loadings matrix is shown in Table 8. The results show that the absolute values of the loading coefficients for all study items on their respective factors are >0.5, indicating a strong association between each item and its corresponding factor. The factor structure is clear and suitable for further analysis and naming.

**Table 8:** Rotated factor load matrix.

Name	Factor load factor				
	Factor 1	Factor 2	Factor 3	Factor 4	Factor 5
AITDA1	0.231	0.195	0.097	0.197	**0.774**
AITDA2	0.195	0.21	0.091	0.125	**0.822**
AITDA3	0.2	0.203	0.138	0.174	**0.807**
AITDA4	0.177	0.219	0.117	0.139	**0.809**
ProF1	0.114	**0.833**	0.07	0.084	0.147
ProF2	0.153	**0.794**	0.073	0.111	0.177
ProF3	0.101	**0.848**	0.074	0.096	0.097
ProF4	0.172	**0.774**	0.109	0.128	0.158
ProF5	0.072	**0.833**	0.076	0.12	0.138
ProF6	0.121	**0.837**	0.046	0.119	0.132
PreF1	0.119	0.094	**0.832**	−0.148	0.089
PreF2	0.087	0.073	**0.832**	−0.061	0.059
PreF3	0.146	0.096	**0.821**	−0.119	0.056
PreF4	0.095	0.07	**0.81**	−0.132	0.09
PreF5	0.106	0.035	**0.808**	−0.156	0.035
PreF6	0.092	0.069	**0.804**	−0.114	0.141
AICT1	**0.792**	0.148	0.103	0.163	0.141
AICT2	**0.816**	0.091	0.101	0.145	0.099
AICT3	**0.818**	0.152	0.164	0.152	0.137
AICT4	**0.824**	0.059	0.072	0.113	0.11
AICT5	**0.782**	0.099	0.093	0.147	0.199
AICT6	**0.825**	0.134	0.081	0.153	0.11
AICT7	**0.788**	0.129	0.12	0.106	0.094
DIC1	0.17	0.133	−0.19	**0.807**	0.123
DIC2	0.152	0.071	−0.165	**0.777**	0.12
DIC3	0.224	0.158	−0.151	**0.78**	0.125
DIC4	0.176	0.135	−0.216	**0.78**	0.126
DIC5	0.169	0.155	−0.068	**0.785**	0.143

Bold values represent factor loadings with absolute values greater than 0.5, indicating that the corresponding item has a strong association with the extracted factor.

#### 5.3.4 Confirmatory factor analysis

Model comparison analysis revealed that the five-factor model (comprising the five constructs AITDA, ProF, PreF, AICT, and DIC) exhibited the best fit, as shown in Table 9. The specific indices are as follows: χ^2^/df = 1.46 (< 3), IFI = 0.986, TLI = 0.984, CFI = 0.986 (all > 0.95), RMSEA = 0.028 (< 0.05). All indicators reached ideal levels. In contrast, the fit of the four-factor, three-factor, two-factor, and single-factor models was significantly weaker: their CFI and TLI were both below 0.90, RMSEA ranged from 0.081 to 0.16, and χ^2^/df also increased substantially (4.714–15.707), indicating that these models had poor fit with the data. Taking all indicators into account, the five-factor model is the only one with good fit, supporting the discriminant validity among the five constructs, AITDA, ProF, PreF, AICT, and DIC, and indicating that they represent five mutually independent and structurally distinct latent variables.

**Table 9:** Results of confirmatory factor analysis.

Model	χ^2^	df	χ^2^ /df	IFI	TLI	CFI	RMSEA
Five-factor model (AITDA, ProF, PreF, AICT, DIC)	496.417	340	1.46	0.986	0.984	0.986	0.028
Four-factor model (AITDA + ProF, PreF, AICT, DIC)	1621.694	344	4.714	0.884	0.873	0.884	0.081
Three-factor model (AITDA + ProF + PreF, AICT, DIC)	3910.864	347	11.271	0.678	0.648	0.677	0.135
Two-factor model (AITDA + ProF + PreF, AICT + DIC)	5481.748	349	15.707	0.536	0.496	0.534	0.161
Single-factor model (AITDA + ProF + PreF + AICT + DIC)	6915.786	350	19.759	0.407	0.358	0.405	0.182

#### 5.3.5 Convergent validity

This study conducted a comprehensive evaluation of the measurement model's quality using composite reliability (CR), average variance extracted (AVE), factor loadings, and Cronbach's alpha. Composite reliability (CR) reflects the internal consistency of each latent variable and is typically required to be >0.7; Average Variance Extracted (AVE) is used to assess convergent validity; a value >0.5 indicates that the latent variable effectively explains the variance of the corresponding measurement scale.

The analysis results show (see Table 10): (1) the standardized loadings of all measurement items ranged from 0.76 to 0.855, all exceeding the recommended standard of 0.70, indicating that each item effectively reflects its corresponding latent variable. (2) The AVE values for the five latent variables (AITDA, ProF, PreF, AICT, DIC) were 0.687, 0.671, 0.649, 0.669, and 0.636, respectively, all exceeding 0.50; the composite reliabilities (CR) were 0.898, 0.924, 0.917, 0.934, and 0.897, respectively, all exceeding 0.70, indicating that the model possesses good internal consistency and convergent validity. (3) The Cronbach's α coefficients for each latent variable ranged from 0.897 to 0.932, all exceeding the acceptance threshold of 0.70, further supporting the scale's excellent reliability. In summary, this measurement model performs well in terms of factor loadings, composite reliability, average variance extracted, and internal consistency reliability. It possesses ideal convergent validity and reliability and is suitable for subsequent structural equation modeling analysis.

**Table 10:** Measurement model.

Variables	Factor load	CR	AVE	Cronbach's α
AITDA	0.76–0.819	0.898	0.687	0.897
ProF	0.78–0.855	0.924	0.671	0.924
PreF	0.779–0.814	0.917	0.649	0.917
AICT	0.769–0.831	0.934	0.669	0.932
DIC	0.787–0.827	0.897	0.636	0.897

Because EFA and CFA were conducted on the same formal sample in the primary analyses, an additional split-half cross-validation was performed to rule out capitalizing on chance. The formal sample was randomly split into two halves (*n*_1_ = 284, *n*_2_ = 284). EFA on the first half again yielded a five-factor structure accounting for 72.1% of the total variance, and CFA on the second half showed acceptable fit (χ^2^/df = 1.42, CFI = 0.975, TLI = 0.973, RMSEA = 0.038); CFA on the first half was similarly acceptable (χ^2^/df = 1.29, CFI = 0.982, TLI = 0.980, RMSEA = 0.032). These cross-validation results are reported in Table 11 and indicate that the five-factor structure is not specific to any single subsample.

**Table 11:** Split-half EFA/CFA cross-validation of the formal sample.

Subsample	*n*	Analysis	Variance explained (%)	χ^2^/df	CFI	TLI	RMSEA
Half A	284	EFA	72.10	—	—	—	—
Half A	284	CFA	—	1.29	0.982	0.980	0.032
Half B	284	CFA	—	1.42	0.975	0.973	0.038

### 5.4 Pearson correlation

According to the results in Table 12, there are significant correlations among all core variables (*p* < 0.001). Specifically, AITDA is significantly positively correlated with ProF (*r* = 0.447), AICT (*r* = 0.453), and DIC (*r* = 0.366), while the positive correlation with PreF is relatively weaker (*r* = 0.240). ProF was significantly positively correlated with DIC (*r* = 0.309) and also positively correlated with AICT (*r* = 0.255). It is worth noting that PreF showed a moderate positive correlation with AICT (*r* = 0.493) but a significant negative correlation with DIC (*r* = −0.262), indicating that individuals with a stronger tendency toward defensive regulation actually had lower DIC scores. Furthermore, AICT showed a significant but weak positive correlation with DIC (*r* = 0.159). From the descriptive statistics, the means of the variables ranged from 4.706 to 5.088, and the standard deviations ranged from 1.260 to 1.342, indicating a reasonable data distribution without obvious ceiling or floor effects. Overall, the direction and strength of correlations among the variables generally align with theoretical expectations, providing preliminary support for subsequent hypothesis testing.

**Table 12:** Pearson correlation coefficients.

Variable	Mean	Standard deviation	AITDA	ProF	PreF	AICT	DIC
AITDA	5.085	1.342	1				
ProF	5.088	1.296	0.447^***^	1			
PreF	4.984	1.260	0.240^***^	0.184^***^	1		
AICT	4.706	1.328	0.453^***^	0.255^***^	0.493^***^	1	
DIC	4.915	1.271	0.366^***^	0.309^***^	−0.262^***^	0.159^***^	1

^*^p < 0.05.

^**^p < 0.01.

^***^p < 0.001.

### 5.5 Hypothesis testing

#### 5.5.1 Main effect testing

Hierarchical regression was used to test the main effects, with the seven control variables entered in the first step and AITDA added in the second step (Table 13). In Model 2, AITDA was significantly positively associated with ProF (β = 0.445, *p* < 0.001), and the model explained 22.0% of the variance (Δ*R*^2^ = 0.194). In Model 4, AITDA was significantly positively associated with PreF (β = 0.228, *p* < 0.001, *R*^2^ = 0.073). In Model 6, AITDA was significantly positively associated with DIC (β = 0.375, *p* < 0.001, *R*^2^ = 0.142). When the mediators were entered simultaneously (Tables 14, 15), AITDA (*b* = 0.349, *p* < 0.001), ProF (*b* = 0.209, *p* < 0.001), and PreF (*b* = −0.393, *p* < 0.001) remained significant (*R*^2^ = 0.307, *p* < 0.001). Among the control variables, gender, age, education, grade, Experience with AI, and training experience were not significantly associated with the core variables in most models; weekly creation time showed a weak negative association with ProF (β = −0.096, *p* < 0.05) and PreF (β = −0.110, *p* < 0.01) before AITDA was entered, but these effects became non-significant after AITDA was included. All VIFs were below 3 (Table 2), indicating that multicollinearity did not distort the estimates. H1, H2, H4, and H5 were supported.

**Table 13:** Results of the main-effects tests.

Variables	ProF (M1)	ProF (M2)	PreF (M3)	PreF (M4)	DIC (M5)	DIC (M6)	DIC (M7)	DIC (M8)
AITDA		0.445^***^		0.228^***^		0.375^***^	0.295^***^	0.460^***^
ProF							0.179^***^	
PreF								−0.375^***^
Gender	−0.068	−0.069	0.035	0.034	−0.036	−0.037	−0.025	−0.024
Age	0.070	0.069	0.081	0.080	0.017	0.016	0.004	0.046
Education	−0.002	0.018	−0.005	0.006	0.017	0.034	0.031	0.036
Grade	0.041	0.052	−0.002	0.004	0.020	0.029	0.020	0.031
Experience with AI	0.025	0.027	0.026	0.027	−0.041	−0.039	−0.044	−0.029
Weekly creation time	−0.096^*^	−0.036	−0.110^**^	−0.079	−0.014	0.037	0.043	0.007
Formal AI training	0.042	0.036	0.028	0.025	0.015	0.010	0.003	0.019
*R* ^2^	0.026	0.220	0.022	0.073	0.005	0.142	0.167	0.272
Adjusted *R*^2^	0.014	0.209	0.010	0.060	−0.008	0.130	0.154	0.260
*F*	2.154	19.678	1.827	5.520	0.390	11.567	12.437	23.188

^*^p < 0.05, ^**^p < 0.01, ^***^p < 0.001.

**Table 14:** Bootstrap results of the mediation analysis.

Effect	Estimate	Boot SE	Boot LLCI	Boot ULCI	Proportion of total effect
Total effect	0.3469	0.0370	0.2742	0.4197	—
Direct effect	0.3424	0.0378	0.2681	0.4167	98.7%
Total indirect effect	0.0045	—	−0.0433	0.0596	1.3%
Indirect effect via ProF	0.0929	0.0222	0.0532	0.1416	26.8%
Indirect effect via PreF	−0.0884	0.0179	−0.1229	−0.0532	−25.5%

**Table 15:** Subgroup heterogeneity analyses.

Group	*n*	AITDA → DIC	AITDA → ProF	AITDA → PreF	ProF → DIC (with mediators)	PreF → DIC (with mediators)
Full sample	568	0.375^***^	0.445^***^	0.228^***^	0.209^***^	−0.393^***^
Bachelor and below	426	0.297^***^	0.448^***^	0.245^***^	0.215^***^	−0.373^***^
Master and above	142	0.514^***^	0.397^***^	0.215^**^	0.212^**^	−0.408^***^
Experience with AI (AI use ≤ 1 year)	243	0.351^***^	0.399^***^	0.212^***^	0.182^**^	−0.402^***^
Experience with AI (AI use > 1 year)	325	0.401^***^	0.502^***^	0.232^***^	0.247^***^	−0.379^***^
Formal AI training (yes)	293	0.372^***^	0.492^***^	0.186^**^	0.187^**^	−0.420^***^
Formal AI training (no)	275	0.374^***^	0.370^***^	0.300^***^	0.244^***^	−0.371^***^
Age 18–23	391	0.352^***^	0.394^***^	0.192^**^	0.189^***^	−0.373^***^
Age 24 and above	177	0.396^***^	0.482^***^	0.288^***^	0.249^***^	−0.420^***^

^*^p < 0.05, ^**^p < 0.01, ^***^p < 0.001.

#### 5.5.2 Mediation effects

Bootstrap mediation analysis with 5,000 resamples was used to test H3 and H6 (Table 14). The direct association between AITDA and DIC was significant [effect = 0.3424, 95% CI (0.2681, 0.4167)], as was the total effect [effect = 0.3469, 95% CI (0.2742, 0.4197)]. The indirect effect through ProF was significantly positive [effect = 0.0929, 95% CI (0.0532, 0.1416)], whereas the indirect effect through PreF was significantly negative [effect = −0.0884, 95% CI (−0.1229, −0.0532)]. These two specific indirect effects were comparable in absolute magnitude and opposite in direction, supporting H3 and H6.

Importantly, the total indirect effect, the algebraic sum of the two specific indirect effects, was 0.0045 [95% CI (−0.0433, 0.0596)], which included zero. Relative to the total effect, the ProF pathway accounted for 26.8% and the PreF pathway for −25.5% of the total effect. The combination of (1) two significant, opposite-signed indirect effects of comparable magnitude, (2) a non-significant total indirect effect, and (3) a significant direct effect satisfies the double-edged sword criteria defined in Section 2.5: the positive and negative pathways are essentially offsetting, and the overall association of AITDA with DIC operates predominantly through the direct pathway.

#### 5.5.3 Moderation effects

Table 16 reports the moderation analyses with mean-centered predictors (unstandardized coefficients). In Model 1, AITDA (*b* = 0.4806, *p* < 0.001) and AICT (*b* = 0.1164, *p* < 0.01) were positively associated with ProF, and the AITDA × AICT interaction was significant (*b* = 0.2088, *p* < 0.001; *R*^2^ = 0.3317, *F* = 27.6495), supporting H7a. In Model 2, AITDA (*b* = 0.0791, *p* < 0.05) and AICT (*b* = 0.5066, *p* < 0.001) were positively associated with PreF, and the interaction was significant (*b* = 0.1867, *p* < 0.001; *R*^2^ = 0.3482, *F* = 29.7494). The positive sign of the interaction on the PreF pathway indicates that AICT strengthened, rather than weakened, the association between AITDA and PreF; H7b was therefore not supported.

**Table 16:** Results of the moderation analyses (unstandardized coefficients).

Type	Variable	ProF (Model 1) b	SE	PreF (Model 2) b	SE
Independent variable	AITDA	0.4806^***^	0.0388	0.0791^*^	0.0373
Moderator	AICT	0.1164^**^	0.0386	0.5066^***^	0.0371
Interaction	AITDA × AICT	0.2088^***^	0.0219	0.1867^***^	0.0211
*R* ^2^		0.3317		0.3482	
*F*		27.6495		29.7494	

^*^p < 0.05, ^**^p < 0.01, ^***^p < 0.001.

Simple-slope analyses (Table 17) clarified the nature of the interactions. For the ProF pathway, the positive association between AITDA and ProF was weaker at low AICT [−1 SD; *b* = 0.2033, *p* < 0.001, 95% CI (0.1183, 0.2883)] and stronger at high AICT [+1 SD; *b* = 0.7578, *p* < 0.001, 95% CI (0.6532, 0.8625)]. For the PreF pathway, the association between AITDA and PreF was significantly negative at low AICT [*b* = −0.1688, *p* = 0.0001, 95% CI (−0.2505, −0.0872)] but significantly positive at high AICT [*b* = 0.3270, *p* < 0.001, 95% CI (0.2264, 0.4275)], with the absolute value of the high-AICT simple slope approximately 1.94 times that of the low-AICT slope. Figures 2, 3 plot the simple slopes with 95% confidence bands.

**Table 17:** Simple-slope analyses of the AICT moderation effects.

Pathway	Level of AICT	*b*	SE	*p*	LLCI	ULCI
AITDA → ProF	Low (−1 SD)	0.2033	0.0433	< 0.001	0.1183	0.2883
	High (+1 SD)	0.7578	0.0533	< 0.001	0.6532	0.8625
AITDA → PreF	Low (−1 SD)	−0.1688	0.0416	0.0001	−0.2505	−0.0872
	High (+1 SD)	0.3270	0.0512	< 0.001	0.2264	0.4275

**Figure 2:**
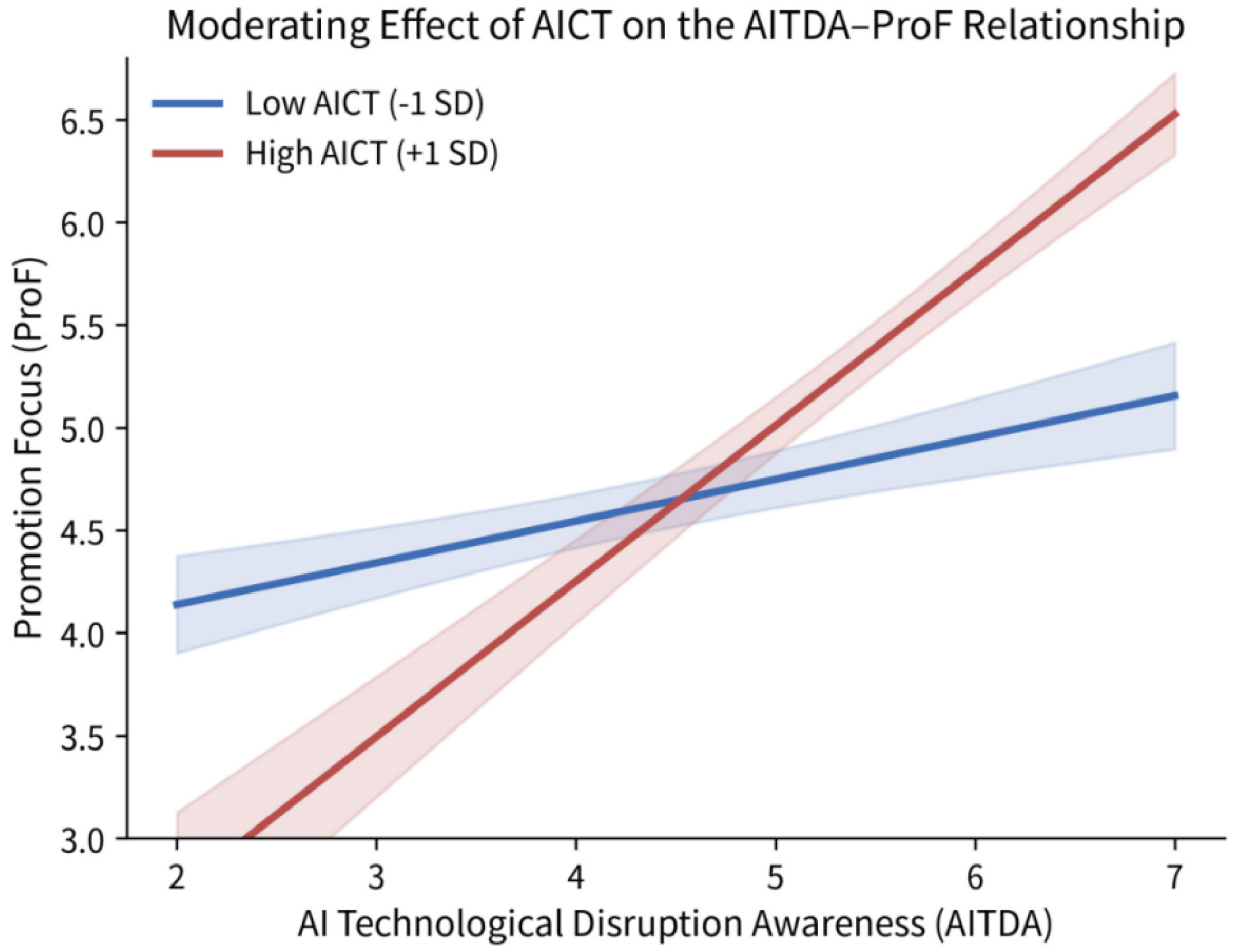
The moderating role of AICT in the relationship between AITDA and ProF. Shaded areas represent 95% confidence bands.

**Figure 3:**
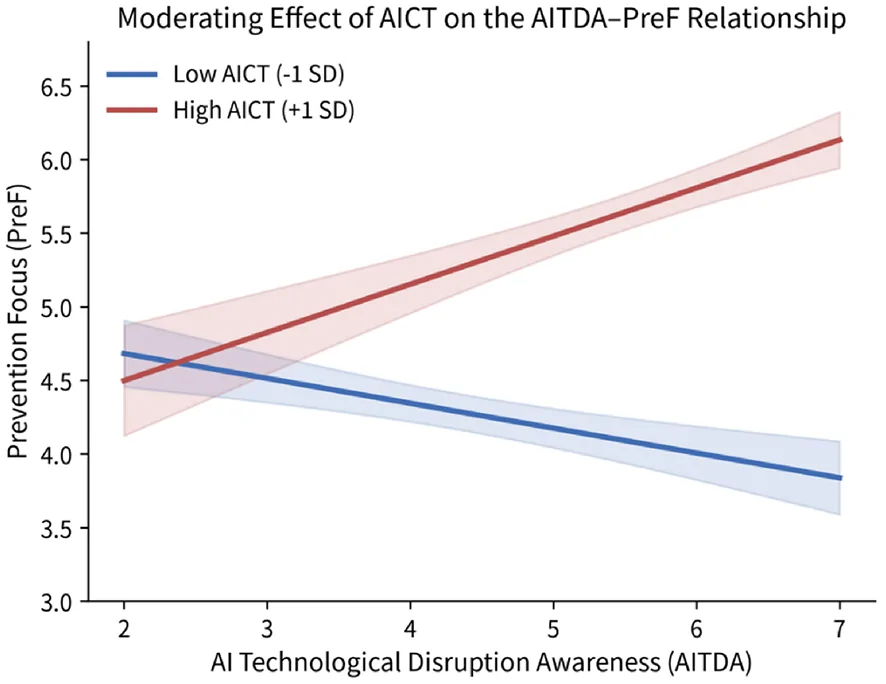
The moderating role of AICT in the relationship between AITDA and PreF. Shaded areas represent 95% confidence bands.

#### 5.5.4 Moderated mediation

To test whether the mediating effects of ProF and PreF were conditional on AICT, conditional indirect effects were estimated at low, mean, and high levels of AICT, together with the index of moderated mediation (Hayes, 2018; Preacher et al., 2007). As shown in Table 18, the indirect effect through ProF was positive and significant at all three levels of AICT [low: 0.042, 95% CI (0.017, 0.081); mean: 0.100, 95% CI (0.063, 0.143); high: 0.158, 95% CI (0.102, 0.218)] and increased monotonically with AICT. The indirect effect through PreF was significantly positive at low AICT [0.066, 95% CI (0.017, 0.107)], non-significant at the mean [−0.031, 95% CI (−0.069, 0.002)], and significantly negative at high AICT [−0.129, 95% CI (−0.187, −0.075)]. The indices of moderated mediation were significant for both pathways [ProF: index = 0.044, 95% CI (0.028, 0.061); PreF: index = −0.073, 95% CI (−0.101, −0.043)], confirming that AICT significantly moderated both mediating mechanisms.

**Table 18:** Conditional indirect effects and indices of moderated mediation.

Mediator	Level of AICT	Conditional indirect effect	SE	LLCI	ULCI
ProF	Low (−1 SD)	0.042	0.016	0.017	0.081
	Mean	0.100	0.022	0.063	0.143
	High (+1 SD)	0.158	0.030	0.102	0.218
PreF	Low (−1 SD)	0.066	0.023	0.017	0.107
	Mean	−0.031	0.017	−0.069	0.002
	High (+1 SD)	−0.129	0.029	−0.187	−0.075
Index of moderated mediation (ProF)		0.044	0.008	0.028	0.061
Index of moderated mediation (PreF)		−0.073	0.015	−0.101	−0.043

^*^p < 0.05, ^**^p < 0.01, ^***^p < 0.001.

#### 5.5.5 Additional robustness analyses

Two additional analyses were conducted to examine the completeness and robustness of the moderated mediation model. First, because the moderation analyses focused on the first stage (AITDA → regulatory focus), the second-stage interactions (ProF × AICT and PreF × AICT on DIC) were also tested. Neither interaction was significant (ProF × AICT: *b* = 0.017, *p* = 0.50; PreF × AICT: *b* = −0.014, *p* = 0.58; Table 19), indicating that the moderating role of AICT operates at the first stage of the mediation model; the significant indices of moderated mediation reported above are consistent with this first-stage pattern.

**Table 19:** Second-stage moderation analyses.

Predictor	DIC model b	SE	*p*
AITDA	0.3493^***^	0.0384	< 0.001
ProF	0.2091^***^	0.0406	< 0.001
PreF	−0.3933^***^	0.0372	< 0.001
AICT	−0.0034	0.0367	0.926
ProF × AICT	0.0170	0.0252	0.500
PreF × AICT	−0.0140	0.0250	0.577

^*^p < 0.05, ^**^p < 0.01, ^***^p < 0.001.

Second, subgroup heterogeneity analyses were conducted to assess the robustness of the core results. The sample was stratified by education level (bachelor and below, ^*^n^*^ = 426; master and above, ^*^n^*^ = 142), AI usage experience ( ≤ 1 year, ^*^n^*^ = 243; > 1 year, ^*^n^*^ = 325), formal AI training (trained, ^*^n^*^ = 293; untrained, ^*^n^*^ = 275), and age (18–23 years, ^*^n^*^ = 391; 24 years and above, ^*^n^*^ = 177). The positive AITDA–DIC relationship, alongside the positive ProF-mediated and negative PreF-mediated pathways, remained significant and directionally stable in every subgroup (Table 15). The AITDA × AICT moderating effects on ProF and PreF were consistently positive and significant across education and AI-experience subgroups (Table 20), supporting the robustness of the moderation pattern.

**Table 20:** Consistency of the AICT moderation effects across education and AI-experience subgroups (standardized coefficients).

Subgroup	*n*	AITDA × AICT → ProF	AITDA × AICT → PreF
Bachelor and below	426	0.222^***^	0.185^***^
Master and above	142	0.191^***^	0.192^***^
Experience with AI (AI use ≤ 1 year)	243	0.210^***^	0.188^***^
Experience with AI (AI use > 1 year)	325	0.209^***^	0.186^***^

^*^p < 0.05, ^**^p < 0.01, ^***^p < 0.001.

Notably, the AITDA–PreF association was weaker among respondents with formal AI training (β = 0.186, ^*^p^*^ < 0.01) compared to those without such training (β = 0.300, ^*^p^*^ < 0.001), suggesting formal AI training can partly reduce threat-based prevention reactions. As all participants majored in design, subgroup analysis by design specialty was not possible, which constitutes a limitation of this research. Overall, these findings verify that our main conclusions are stable across demographic and AI-experience subgroups.

#### 5.5.6 Summary of hypothesis testing

Table 21 summarizes the results for all hypotheses. H1–H6 were supported, H7a was supported, and H7b was not supported (the moderation was significant but in the opposite direction).

**Table 21:** Summary of hypothesis-testing results.

Hypothesis	Path	Key effect	95% CI	Result
H1	AITDA → ProF	β = 0.445^***^		Supported
H2	ProF → DIC	*b* = 0.209^***^		Supported
H3	AITDA → ProF → DIC	Indirect = 0.0929	[0.0532, 0.1416]	Supported
H4	AITDA → PreF	β = 0.228^***^		Supported
H5	PreF → DIC	*b* = −0.393^***^		Supported
H6	AITDA → PreF → DIC	Indirect = −0.0884	[−0.1229, −0.0532]	Supported
H7a	AITDA × AICT → ProF	*b* = 0.2088^***^; index = 0.044	[0.028, 0.061]	Supported
H7b	AITDA × AICT → PreF	*b* = 0.1867^***^; index = −0.073	[−0.101, −0.043]	Not supported (opposite direction)

^*^p < 0.05, ^**^p < 0.01, ^***^p < 0.001.

## 6 Discussion

### 6.1 The double-edged sword mechanism of AITDA

This study examined whether AITDA is associated with design students' DIC through two parallel motivational pathways with opposite signs. The results support the hypothesized double-edged pattern: AITDA was significantly positively associated with ProF, which in turn was positively associated with DIC (the “opportunity–proactive” pathway), while AITDA was simultaneously significantly positively associated with PreF, which was negatively associated with DIC (the “threat–inhibitory” pathway). The two specific indirect effects were comparable in absolute magnitude (0.0929 vs. 0.0884) and opposite in direction, each accounting for approximately one quarter of the total effect (26.8% and −25.5%), whereas the total indirect effect was essentially zero (0.0045, 95% CI [−0.0433, 0.0596]) and the direct effect remained substantial (0.3424). This pattern two significant opposing indirect paths, a null total indirect effect, and a strong direct effect is exactly the configuration predicted by the “symmetrical double-edged sword” account defined in Section 2.5, and it generalizes to the design-education context the findings of Zheng et al. (2025), Shi et al. (2026), and Zhu et al. (2025) on the coexistence of opportunity and threat appraisals in AI disruption.

Substantively, the results indicate that the same external signal awareness that AI may reshape design work can be psychologically constructed in two very different ways. Students who appraise AI disruption as an opportunity develop a growth-oriented motivational state that supplies the cognitive resources for challenging goal-setting, exploratory trial-and-error, and novel solution generation. Students who appraise the same disruption as a threat enter a defensive state in which cognitive resources are diverted to risk monitoring and loss prevention, constraining breakthrough and original conceptual exploration. Because the two pathways largely offset each other at the aggregate level, the net indirect association of AITDA with DIC is negligible; the overall association is carried by the direct pathway. This does not mean that the mediating mechanisms are unimportant on the contrary, the offsetting pattern reveals that the motivational consequences of AITDA are highly heterogeneous across students and cannot be captured by a single total effect (see also Dong et al., 2025). Viewed through the lens of effect proportions, the two pathways are approximately symmetrical (26.8% vs. −25.5%), which strengthens the interpretation of a genuine balancing mechanism rather than a dominant unidirectional effect. It also implies that educational interventions should not aim simply to increase or decrease students' awareness of AI disruption, but rather to shape how that awareness is appraised and translated into motivational states.

### 6.2 The moderating role of AICT

The results support H7a: AICT significantly strengthened the positive association between AITDA and ProF. The simple slope of AITDA on ProF increased from 0.2033 (low AICT) to 0.7578 (high AICT), and the conditional indirect effect through ProF increased from 0.042 to 0.158 across levels of AICT, with a significant index of moderated mediation [0.044, 95% CI (0.028, 0.061)]. Students with higher AICT are better able to analyze technological disruption rationally and dialectically, to identify and seize the opportunities embedded within it, and to convert perceived challenges into motivational drive consistent with Zhang and Zong (2019) and Wang and Zhang (2026).

The results did not support H7b. Instead of weakening the association between AITDA and PreF, AICT strengthened it: the simple slope reversed from −0.1688 (low AICT) to 0.3270 (high AICT), and the conditional indirect effect through PreF shifted from significantly positive at low AICT (0.066) to significantly negative at high AICT (−0.129), with a significant index of moderated mediation [−0.073, 95% CI (−0.101, −0.043)]. This reversal, although contrary to the buffer hypothesis, is consistent with the alternative theoretical account anticipated in Section 3.3. For students with high AICT, the prevention focus activated by AI disruption is less likely to reflect emotional anxiety or ignorance-based avoidance (Hansson et al., 2017; Bok, 2006) and more likely to represent a rational, responsibility-driven vigilance grounded in an understanding of AI's limitations, biases, ethical controversies, and social impacts (Schiavo et al., 2024; Walter, 2024; Helal et al., 2025). In this view, high-AICT students “grip the hilt of the double-edged sword”: they maintain necessary caution and ethical boundaries while remaining open to exploration, rather than retreating from innovation. The finding that AICT transforms the nature rather than merely the strength of the AITDA–PreF association qualifies the common assumption that critical thinking uniformly buffers threat reactions, and it extends recent critical-thinking-in-the-AI-era research (Gerlich, 2025; Ji et al., 2025; Melisa et al., 2025) by showing that the moderating pattern depends on which motivational pathway is considered.

This unexpected result also offers a substantive theoretical increment beyond the existing risk-perception literature. Prior research has largely assumed that critical thinking reduces technology-related anxiety and threat appraisals (e.g., studies of AI literacy and acceptance). Our results suggest a more differentiated picture: critical thinking about AI does not dissolve risk perception; it converts diffuse, emotion-driven anxiety into a reasoned, ethically anchored caution. For design students, this distinction matters because reasoned caution can coexist with and even support innovative behavior when it is channeled into ethical scrutiny and quality assurance, whereas emotional anxiety suppresses exploration. The reversal of the simple slope (from negative to positive) demonstrates that the same construct can have qualitatively different regulatory consequences depending on the individual's cognitive resources, a nuance that has rarely been documented in design-education research.

Taken together, the moderated mediation results indicate that AICT amplifies the positive pathway and, at high levels, converts the negative pathway into a smaller, rational-vigilance form that is less detrimental to innovation. In practical terms, the beneficial role of AICT operates by simultaneously strengthening opportunity-driven motivation and reshaping defensive motivation, rather than by eliminating defensive motivation altogether. The non-significant second stage interactions further indicate that these moderating effects occur when AICT shapes the interpretation of AI disruption (first stage), not when regulatory focus is translated into innovation outcomes (second stage).

### 6.3 Theoretical contributions

This study offers four theoretical contributions. First, it extends RFT from the workplace and management settings where it has most often been applied to the under-examined domain of design education and educational technology. By operationalizing regulatory focus as a situationally activated state in the AI-design context–rather than as a chronic trait and by integrating the appraisal logic of the transactional stress model (Lazarus and Folkman, 1984), the study clarifies how external technological change is translated into individual motivational states, opening a new theoretical perspective on the cultivation of innovative capabilities in the technological age (see also Ma and Zhang, 2025; Shi et al., 2026).

Second, the study empirically validates the double-edged sword effect of AITDA with a dual-path model and quantifies the balance between the two pathways. The near-null total indirect effect combined with two significant opposing indirect effects provides a clear, data-based criterion for what a “double-edged sword” means in this context, going beyond the simple listing of positive and negative findings that characterizes much of the existing literature (Dong et al., 2025; Zheng et al., 2025). The effect-proportion analysis (26.8% vs. −25.5%) shows that the two pathways are approximately symmetrical, which strengthens the interpretation of a genuine balancing mechanism.

Third, the study identifies a novel moderating role of AICT, particularly its reshaping of the prevention pathway. AICT not only reinforces the transformation of disruption awareness into promotion focus, as expected, but also changes the nature of the prevention focus activated by AI disruption: for high–AICT students, the resulting prevention orientation reflects rational, ethics-based prudence rather than blind, anxiety-driven avoidance. This finding challenges the purely negative interpretation of prevention focus in innovation research and deepens the theoretical implications of the double-edged sword effect by showing that the same psychological pathway can have different meanings depending on the individual's cognitive resources (Schiavo et al., 2024; Walter, 2024).

Fourth, the study contributes a scenario-expansion account of RFT in the AI-design context. Unlike most RFT applications, which treat regulatory orientation as a stable disposition (e.g., Wu and Zhang, 2024), the present findings demonstrate that a technology-related situational cue–AITDA–can temporarily activate promotion and prevention orientations among students, supporting the state-activation account of RFT (Crowe and Higgins, 1997; Scholer et al., 2019) in an educational technology setting. Moreover, the moderated-mediation results specify a boundary condition of this state-activation process: the motivational meaning of the activated prevention orientation depends on the individual's AICT. In other words, the same situational activation can lead to qualitatively different motivational trajectories, which enriches RFT's explanatory power for technology-shock contexts and provides a theoretical basis for differentiated pedagogical intervention.

### 6.4 Implications for practice

The findings support several practical recommendations for design education. First, design educators should systematically cultivate students' AICT rather than teaching AI tools as mere operational skills. Because AICT simultaneously strengthens the opportunity pathway and transforms the threat pathway into rational vigilance, placing AICT development at the core of design curricula helps students “grasp the hilt of the double–edged sword”: they can identify technological opportunities and pursue them proactively while maintaining informed caution about AI's limits, biases, and ethical risks. Courses and workshops should therefore train students in algorithmic evaluation (verifying and questioning AI outputs), ethical reflection (responsibility, bias, intellectual property), and creative verification (iteratively integrating AI suggestions with domain knowledge).

Second, the heterogeneity of the motivational consequences of AITDA calls for differentiated guidance rather than one-size-fits-all training. For students with lower AICT, who are more likely to fall into anxiety-driven avoidance under AI disruption, educators should provide structured support that reduces threat appraisal: scaffolding tasks that let students experience AI as an enabling partner, showcasing positive cases of human–machine co-creation, and providing timely recognition of progress and creative achievements to reinforce promotion-oriented motivation. For students with higher AICT, whose prevention focus reflects rational responsibility, educators should channel this vigilance into design ethics and quality assurance practices (e.g., AI-output auditing, bias checking, and responsible-innovation protocols), so that caution supports rather than suppresses innovation.

Third, the subgroup results suggest that formal AI training may attenuate threat-based prevention reactions (the AITDA–PreF association was weaker among trained than untrained students). Design programs should therefore introduce structured AI training early and progressively, rather than leaving students to encounter AI disruption without preparation. At the same time, because the core double-edged pattern and the moderating role of AICT were stable across education levels, AI-experience groups, and age groups, the differentiated guidance should primarily follow students' AICT level and AI experience rather than demographic categories.

Fourth, curriculum and project design should deliberately create contexts that steer appraisals of AI disruption toward challenge rather than threat. Setting challenging, future-oriented human–machine collaborative design tasks; framing AI as a partner for realizing creative ideals; and building a safe, failure-tolerant classroom culture can help students interpret technological change as an opportunity. At the same time, institutions should initiate open dialogue on AI disruption to help students rationally analyze the nature of technological change, clarify the core competencies required of future designers, and transform vague threat perceptions into clear understanding and proactive strategies.

### 6.5 Limitations and future research

Several limitations should be acknowledged. First, the cross-sectional, single-wave design precludes causal inference; the results should be interpreted as associations. Although the hypothesized ordering of variables is theoretically grounded, longitudinal tracking or experimental designs (e.g., priming of opportunity vs. threat appraisals) are needed to test causal chains. Second, the reliance on self-report measures introduces potential common method bias and self-report bias. Although procedural remedies were applied and both Harman's test and the latent method-factor test indicated that common method bias is unlikely to be severe, the first factor accounted for 31.54% of the variance–close to the 40% heuristic–and self-report assessments may overstate the true associations. Third, the study lacks objective indicators of innovation: DIC was measured through self-assessment, and future research should incorporate faculty evaluations, objective assessments of design portfolios, or multi-wave measurement with temporal separation. Fourth, the convenience and snowball sampling from Chinese universities limits the generalizability of the findings; the sample is not a probability sample, non-response bias cannot be fully ruled out (no time-stamped data were available to compare early and late responders), and cross-cultural and cross-disciplinary validation is needed. Fifth, all respondents were design students in China, and the sample was homogeneous in design discipline, which prevented subgroup analyses by design major; cultural and disciplinary boundary conditions of the model remain to be tested in other national and disciplinary contexts. Sixth, the second-stage moderation effects were non-significant, indicating that AICT moderates the first stage only; future research could examine whether other individual or contextual factors (e.g., self-efficacy, peer climate, institutional support) moderate the second stage. Finally, other individual traits (e.g., openness, growth mindset), situational factors (e.g., peer group atmosphere), and unmeasured confounders (e.g., prior innovation foundation, general AI proficiency partially proxied here byExperience with AI) that may influence the mechanisms should be incorporated into future models; multi-level factors such as teachers, schools, and industry ecosystems, which jointly shape the pathways for cultivating design innovation, also warrant future investigation.

Concrete directions for future research include: (1) AICT teaching-intervention tracking experiments that test whether targeted AICT training changes both the strength and the nature of the AITDA–regulatory-focus pathways over time, with pre-post measurement and control groups; (2) cross-national and multi-specialty comparisons to establish the generalizability of the double-edged pattern, including design-major subgroup comparisons where feasible; (3) extension of the moderated mediation model to include second-stage moderators and additional mediators; (4) incorporation of objective indicators of innovation, such as teacher ratings and evaluated design works, to complement self-reported DIC; (5) experience-sampling or diary designs that capture the momentary activation of regulatory focus in real design tasks; and (6) multi-level models examining the roles of teachers, institutions, and industry ecosystems.

## 7 Conclusion

Drawing on regulatory focus theory, this study examined how design students' AI technological disruption awareness is associated with their design innovation competence through two parallel motivational pathways and the moderating role of AI critical thinking. In response to the first research question, AITDA was positively associated with DIC directly. In response to the second, AITDA was simultaneously associated with DIC through a positive promotion-focus pathway and a negative prevention-focus pathway; the two indirect effects were comparable and opposite in direction, yielding a non-significant total indirect effect and thereby confirming a double-edged sword pattern. In response to the third, AICT strengthened the positive pathway and unexpectedly strengthened rather than weakened the negative pathway, with the index of moderated mediation significant for both. Theoretically, the study extends regulatory focus theory to technology-induced change, integrates it with the transactional model of stress and coping, and reveals the double-edged moderating role of AI critical thinking; methodologically, it corrects the mediation estimates and provides conditional, second-stage, and subgroup evidence; and practically, it suggests that design education should cultivate AI critical thinking while guiding students to channel both growth-oriented and reasoned, responsible responses toward positive adaptation and innovation. Because the findings are based on cross-sectional, self-report data, they should be read as associations rather than causal claims, and longitudinal and experimental work is needed to confirm them.

## Data Availability

The original contributions presented in the study are included in the article/supplementary material, further inquiries can be directed to the corresponding author/s.
